# Immune Checkpoint Inhibitors and Atherosclerotic Vascular Events in Cancer Patients

**DOI:** 10.3389/fcvm.2021.652186

**Published:** 2021-05-28

**Authors:** Alessandro Inno, Andrea Chiampan, Laura Lanzoni, Matteo Verzè, Giulio Molon, Stefania Gori

**Affiliations:** ^1^Medical Oncology, Istituto di Ricovero e Cura a Carattere Scientifico (IRCCS) Ospedale Sacro Cuore Don Calabria, Verona, Italy; ^2^Cardiology Department, Istituto di Ricovero e Cura a Carattere Scientifico (IRCCS) Ospedale Sacro Cuore Don Calabria, Verona, Italy; ^3^Medical Direction, Istituto di Ricovero e Cura a Carattere Scientifico (IRCCS) Ospedale Sacro Cuore Don Calabria, Verona, Italy

**Keywords:** arterial thrombosis, ischemic stroke, myocardial infarction, atherosclerosis, PD-L1, PD-1, CTLA-4, acute vascular events

## Abstract

In clinical trials and meta-analysis, atherosclerotic vascular events (AVEs) during treatment with immune-checkpoint inhibitors (ICIs) have been reported with low incidence. However, preclinical data suggest that these drugs can promote atherosclerosis inflammation and progression of atherosclerosis plaques, and there is now growing and convincing evidence from retrospective studies that ICIs increase the risk of atherosclerotic vascular events including arterial thrombosis, myocardial infarction and ischemic stroke. Prospective studies are needed to increase knowledge on long-term effect of ICIs or their combinations with other cardio-toxic drugs, but in the meantime a careful assessment and optimization of cardiovascular risk factors among patients treated with ICIs is advisable.

## Introduction

Immune checkpoint inhibitors (ICIs) have extended survival across many tumor types and their use in cancer treatment has been increasing over time (1). ICIs are monoclonal antibodies targeting immune checkpoints, proteins that play a negative regulatory function within the immune system (2). Currently approved ICIs are directed against the cytotoxic T-lymphocyte-associated protein 4 (CTLA-4), the programmed death 1 (PD-1) and one of its ligands, the programmed death ligand 1 (PD-L1) (3). By binding their target, ICIs release the brakes that cancer cells place on the immune system, thus unleashing the immune cells against the tumor. On the other hand, however, ICIs are characterized by a peculiar toxicity profile consisting of immune-related adverse events (irAEs) that may potentially affect any organ or system, including the cardiovascular system (4, 5).

Initially, atherosclerotic vascular events (AVEs) such as arterial thrombosis, coronary artery disease (CAD), acute coronary syndrome (ACS), myocardial infarction (MI) and ischemic stroke were not specifically recognized as irAEs and therefore not usually considered as a possible toxicity of ICIs. However, there is now growing preclinical and clinical evidence suggesting a possible correlation between ICIs and AVEs. In the present review we summarize and discuss the available literature on this topic.

## Materials and Methods

For the present review, the PubMed database was searched from the inception to 31st January, 2021, using the following terms: (“CTLA-4” OR “PD-1” OR “PD-L1” OR “immune checkpoint^*^” OR “immune checkpoint inhibitor^*^” OR “anti-CTLA-4” OR “anti-PD-1” OR “anti-PD-L1” OR “ipilimumab” OR “tremelimumab” OR “nivolumab” OR “pembrolizumab” OR “atezolizumab” OR “durvalumab” OR “cemiplimab”) AND (“atherosclerosis” OR “atherosclerotic plaque” OR “vascular event^*^” OR “arterial thrombosis” OR “coronary artery disease” OR “acute coronary syndrome” or “myocardial infarction” OR “ischemic stroke”).

## Immune System and Atherosclerosis

Atherosclerosis is a complex disease process initiated by the retention in the arterial walls of low-density lipoprotein (LDL)-cholesterol, that may undergo oxidative modification leading to the formation of oxidide LDL (oxLDL). The accumulation of oxLDL may elicit an innate inflammatory response with the recruitment of circulating monocytes that, infiltrating arterial walls, differentiate into macrophages and at a later stage transform into foam cells that eventually die creating a core area in the plaque that consists of necrotic cells and cholesterol crystals (6).

As the atherosclerotic plaque grows, accumulation of immune cells and particularly T cells occurs at the shoulder regions of the lesion. In this context of chronic inflammation, adaptive immune response plays a crucial role, and T-cells that recognize autoantigenic components of LDL regulate plaque development (6). Particularly, T helper type 1 cells (Th1) produce interferon-γ (IFNγ), which promotes macrophage activation and counteracts cap formation by enhancing collagen degradation and inhibiting smooth muscle cell proliferation, thus leading to vulnerable plaques that on hemodynamic assaults may undergo rupture with endothelial dysfunction and thrombus apposition, thus leading to acute events such as myocardial infarction or stroke (7). On the other hand, regulatory T cells (Treg) limit Th1 responses in the plaque and T helper type 17 cells (Th17) promotes plaque stability by enhancing collagen deposition, leading to increased cap formation (6).

T cell functions are finely regulated by immune checkpoints, including CTLA-4 and PD-1 that represent now targets for cancer immunotherapy. CTLA-4 is mainly involved in the priming phase of T cell activation, whereas PD-1 is involved in the effector phase (3). When the naïve T cells recognize the antigens presented by antigen presenting cells (APCs) in the lymph nodes through their T cell receptor (TCR), to be fully activated they need a second costimulatory signal that is provided by the interaction of CD28 expressed on the T cell membrane with the B7-1 (CD80) or B7-2 (CD86) molecules on the surface of APCs. CTLA-4 is upregulated on the T-cell membrane shortly after T-cell activation, and the binding of CTLA-4 to B7 molecules provides inhibitory signals for the T cell and induces Treg responses, thereby limiting inflammation and preventing autoimmunity. PD-1 inhibitory receptor is expressed by exhausted T cells after long-term exposure to antigens and exerts a negative regulation when it binds to one of its ligands, PD-L1, or PD-L2, present in inflamed tissues such as atherosclerotic lesions, or tumor microenvironment.

## Preclinical Studies

Results from preclinical studies suggest that the blockade of CTLA-4 or PD-1/PD-L1 pathway plays a relevant role in promoting progression of the atherosclerotic lesions (Table 1) (11, 12). A short-term treatment with an anti-CTLA-4 antibody led to endothelial activation, accelerated the progression of atherosclerosis by inducing a predominantly T cell-driven inflammation, and resulted in the formation of plaques with larger necrotic cores and less collagen in an *in vivo* atherosclerosis experimental model based on hypercholesterolemic, low-density lipoprotein receptor (LDL-R) knock-out mice (*ldlr*^−/−^ mice) (11).

**Table 1:** Preclinical studies.

**References**	**Model**	**Main findings**
Gotsman et al. (8)	hypercholesterolemic *pdl^−/−^ ldlr^−/−^* mice and *ldlr^−/−^* controls	PD-L1/2 deficiency led to: • increased atherosclerotic burden throughout the aorta • increased numbers of lesional CD4^+^ and CD8^+^ T-cells. Increase numbers of activated CD+ T-cells in iliac lymphadenopathy • higher levels of serum TNF-α • more effective APCs in activating CD4^+^ T cells
Bu et al. (9)	hypercholesterolemic *pdl^−/−^ ldlr^−/−^* mice, *ldlr^−/−^* mice treated with anti-PD-1, and *ldlr^−/−^* controls	PD-L1/2 deficiency led to: • larger atherosclerotic lesions with more abundant CD4^+^ and CD8^+^ T-cells and macrophages • higher levels of serum TNF-α • more proliferation of iliac lymph nodes T-cells to oxLDL • more cytotoxic activity of CD8^+^ T-cellsAnti-PD-1 led to: • increased plaque inflammation with more lesional T-cells • more activated T-cells in paraortic lymph nodes
Cochain et al. (10)	hypercholesterolemic *pdl^−/−^ ldlr^−/−^* mice and *ldlr^−/−^* controls	PD-L1/2 deficiency led to: • increased systemic CD4^+^ and CD8^+^ T-cell activation • expansion of both pro-atherogenic IFNγ-secreting T_H1_ and atheroprotective Foxp3^+^ T_regs_ • massive infiltration of T cells in atherosclerotic lesions • aggravated hypercholesterolemia and exacerbated atherosclerotic lesion development
Poels et al. (11)	Hypercholesterolemic *ldrl^−/−^* mice, treated with anti-CTLA-4 or control.	Anti-CTLA-4 led to: • 2.0-fold increase in the plaque area in the aortic area • more advanced morphological phenotype and an increased T cell/macrophage ratio in the plaque • activated T-cell profile in the blood and lymphoid organs

**Figure 1:**
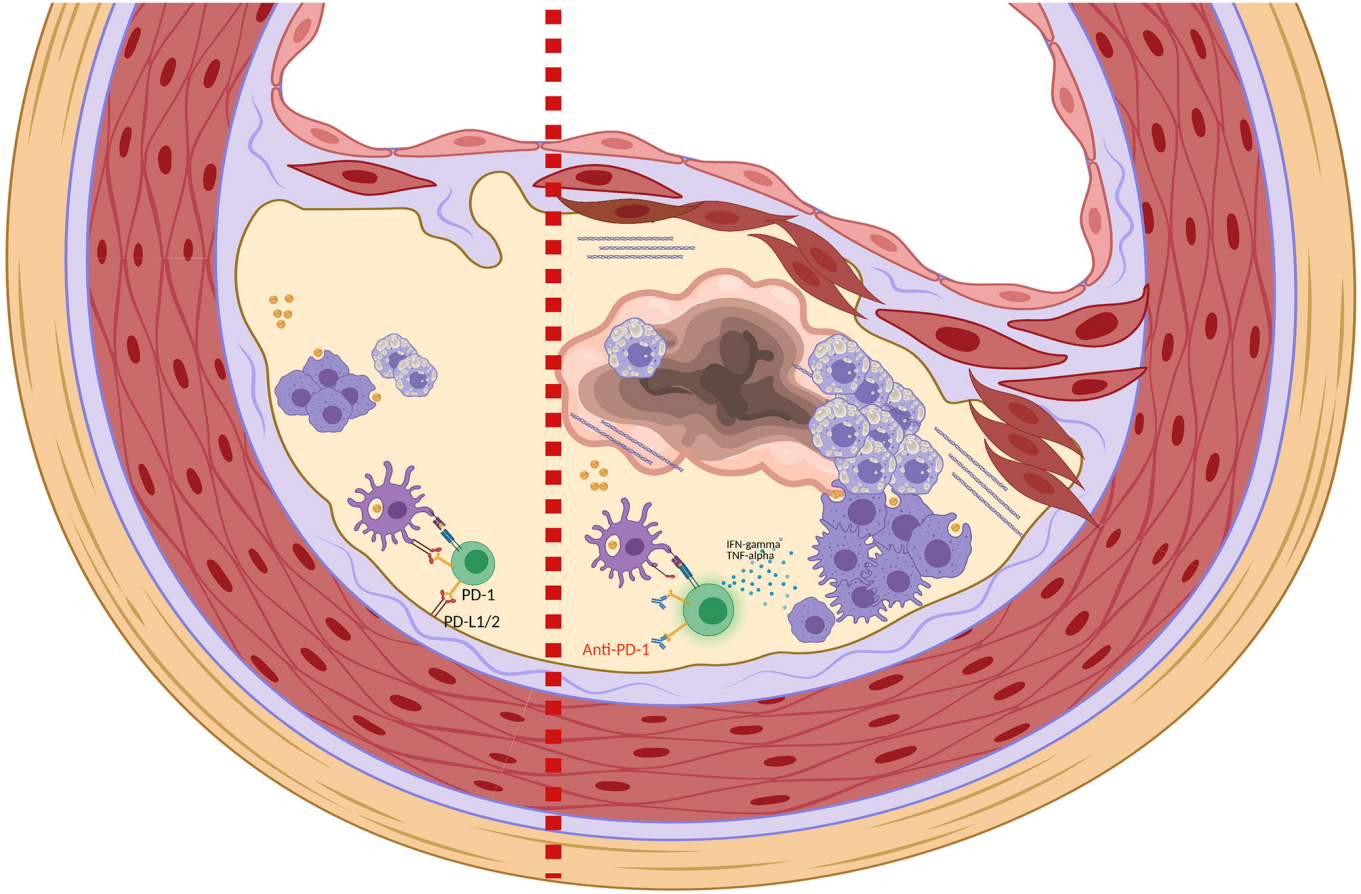
Role of PD-1 and PD-1 blockade in the homeostasis of atherosclerotic plaque. Left side: Binding of PD-1 expressed on T-cells with PD-L1/PD-L2 expressed on DCs and in microenvironment contribute to the inactivation of T-cells and to the maintenance of an immunosuppressive plaque microenvironment. Right side: Anti-PD-1 leads to T-cells activation with production of proinflammatory cytokines such as TNF-α and IFNγ. TNF-α and IFNγ promote smooth muscle cell proliferation, collagen deposition and activation of macrophages that increase the phagocytosis of LDL with transformation into foam cells (8, 9). These changes in the plaque structure ultimately lead to the formation of a necrotic core and plaque instability (Picture created with Biorender.com).

Regarding PD-1/PD-L1, several preclinical studies showed that PD-1 exerts significant atheroprotective effects, PD-1/PD-L1 pathway downregulates the proatherogenic Tcell response, and PD-1/PD-L1 deficiency promotes atherosclerosis (Figure 1) (8–10, 12). Particularly, an *in vivo* study showed that *ldlr*^−/−^ mice receiving high-cholesterol diet for 10 weeks had increased PD-L1 and B7-1 expression in dendritic cells (DCs) from the iliac lymph nodes, and increased PD-L1 and PD-L2 expression in peritoneal macrophages, compared with mice receiving control-diet (8). In this study, modified mice lacking for both LDL-R and PD-L1/2 genes (*pdl*^−/−^ *ldlr*^−/−^ mice) developed a significant increase in the aortic atherosclerotic burden after 10 weeks of high-cholesterol diet, with a 2-fold increase of plaques in aortic root and a 3-fold increase of plaques in aortic arch and descending aorta, when compared with the control group (*ldlr*^−/−^ mice). In comparison with the control group, *pdl*^−/−^ *ldlr*^−/−^ mice had also increased smooth muscle cells and collagen deposition in the plaques, increased CD4^+^ and CD8^+^ T-cells and macrophages in the intima, increased CD4^+^ T-cells with activation phenotype (CD25^+^CD62L^lo^) in the iliac lymph nodes, and increased serum TNF-a levels. Furthermore, macrophages and DCs taken from *pd1*^−/−^ *ldlr*^−/−^ mice led to increased CD4^+^ T cell proliferation *in vitro* as compared with those taken from control mice (8). A subsequent study reported that the administration of an anti-PD-1 antibody to *ldlr*^−/−^ mice fed with high-cholesterol diet resulted into enhanced lesional inflammation characterized by increased CD4^+^ and CD8^+^ T-cells, associated with more CD44^+^ and IFN-g-producing CD4^+^ and CD8^+^ T-cells in the iliac lymph nodes, as compared with *ldlr*^−/−^ mice not receiving the anti-PD-1 antibody (9). Overall, these data suggest that PD-1/PD-L1 axis has an important role in downregulating atherosclerosis by limiting APC-dependent T-cell activation, and that PD-1/PD-L1 blockade may contribute to atherosclerosis progression in murine models through increased activation of CD4^+^ and CD8^+^ T-cells.

Anti-CTLA-4 and anti-PD-1/PD-L1 antibodies may alter the composition of atherosclerotic plaque not only in experimental murine models but also in humans. In fact, an autopsy study evaluating the inflammatory infiltrate in coronary artery atherosclerotic plaques from cancer patients reported a significant increase in T-cells/macrophages ratio in patients who had been recently treated with ICIs compared to those who had not treated with ICIs (13). It was postulated that the ICI-induced switch from a macrophage-predominant to a lymphocyte-predominant plaque may lead to atherosclerosis progression and plaque instability (14), although the lymphocytes/macrophages ratio may not represent the best parameter to describe the quality of the immune infiltrate in atherosclerotic plaques, given that different types of lymphocytes may exert different roles in the atherosclerosis progression (6). In fact, a study evaluating carotid plaques from 29 patients undergoing endarterectomy reported a higher number of CD4^+^ and CD8^+^ T cells but a lower number of Tregs in unstable lesions compared with stable lesions (15). More preclinical and translational studies aiming to obtain a better qualitative characterization of the immune infiltrates in atherosclerotic plaques after ICIs exposure would be helpful to elucidate the role of ICIs on the atherosclerotic process.

## Case Reports

Several cases of AVEs during treatment with ICIs in cancer patients have been reported (Table 2). In 2017, a case of ACS due to right coronary artery occlusion was described in a patient with metastatic NSCLC achieving a complete response to the anti-PD-1 antibody nivolumab (17). Although the patient had pre-existing cardiovascular factors including dyslipidemia treated with atorvastatin and smoking history, the concomitant development of multiple irAEs including fever, erythema multiforme, thyroid dysfunction, and interstitial pneumonia suggested a role for nivolumab in the development of ACS. Since then, other reports of ACS possibly related to anti-PD-1 have been published (18–20). Particularly, one patient with metastatic giant cell bone tumor treated with pembrolizumab experienced 2 subsequent events of non-ST elevation myocardial infarction (NSTEMI), with finding at serial coronary angiography of progressive stenosis of the left circumflex artery 2 months apart (20). Such a rapid progression of atherosclerosis is consistent with data deriving from mice models indicating a role for PD-1 blockade in atherosclerosis progression (9). Although atherosclerosis progression remains one the most likely underlying mechanisms of ICI-related ACS or MI, other speculations on the pathogenesis include a coronary spasm possibly secondary to systemic inflammatory response syndrome induced by ICIs (18), or T cell mediated coronary vessel vasculitis in the absence of atherosclerosis (5).

**Table 2:** Case reports.

**References**	**Age, sex, cancer**	**ICI**	**CV risk factor**	**AVE**	**Associated irAEs**	**Tumor response**	**Treatment**	**Outcome**
Boutros et al. (16)	71 yo, M, stage IV melanoma	Pembrolizumab	NR	Arterial thrombosis (left leg)	Diabetes	Partial response	arterial embolectomy, foot amputation	Resolution ICI discontinued
	69 yo, F	Pembrolizumab	Dyslipidemia	Pulmonary embolism with bilateral lobar artery thrombosis	–	Completer response	Anticoagulation was initiated and intravenous thrombolysis	Resolution ICI discontinued
	78 yo, M	Pembrolizumab	NR	Arterial thrombosis (right common iliac artery, external and internal iliac Arteries and peripheral bilateral artery disease)	–	Partial response	Antiplatelet drug; patient refused bypass graft	NR ICI discontinued
	53 yo, M	Ipilimumab/Nivolumab	History of smoking	Stenosis of the left subclavian artery related to an atherosclerotic plaque with a floating arterial thrombus	Pneumonitis	Progressive disease	Anticoagulant, antiplatelet, and statin therapy	NR ICIs discontinued
Tomita et al. (17)	61 yo, M, stage IV NSCLC,	Nivolumab	Dyslipidemia, history of smoking	ACS	Thyroiditis, erythema multiforme, pneumonitis	Complete response	Stenting	Resolution
Nykl et al. (18)	71 yo, M, stage IV NSCLC,	Pembrolizumab	–	Temporary coronary spasm with inferior STEMI	Systemic inflammation response syndrome	NR	Acetylsalicylic acid, clopidogrel, heparin, and vasopressor support	Resolution ICI restarted
Ferreira et al. (19)	60 yo, F, stage IV NSCLC	Nivolumab	History of smoking	Temporary coronary spasm with ACS	–	Stable disease	Acetylsalicylic acid, clopidogrel, verapamil	Resolution ICI discontinued
	72 yo, M, stage IV melanoma	Nivolumab	NR	ACS	NR	–	Oxygen, nitrates, bisoprolol, eplerenone, furosemide	Death
	53 yo, F, Hodgkin Lymphoma	Nivolumab	NR	Fugitive repolarization disorders	NR	Partial response	Steroids	NR
Kwan et al. (20)	71 yo, M, stage IV giant cell bone tumor	Pembrolizumab	Hypertension, type 2 diabetes, history of smoking, peripheral artery disease	NSTEMI	Primary biliary cholangitis	Stable disease	Atherectomy, stenting, acetylsalicylic acid, clopidogrel, and atorvastatin	Resolution

In 2017, four cases of arterial thrombosis in cancer patients treated with anti-PD-1 antibodies were described (16). One of these patients underwent Fogarty arterial embolectomy, and histological examination revealed that CD8^+^ T cells were present in the superficial arterial wall, and the thrombus fragments contained large aggregates of entrapped leucocytes, including numerous neutrophils, monocytes and macrophages, with rare T cells and B cells, but no tumor cells were detected. PD-L1 was not expressed in entrapped leucocytes or vascular lining cells (9). The presence of CD8^+^ T-cells in the arterial wall of this patient is consistent with preclinical findings of an increased amount of CD8^+^ T-cells in atherosclerotic lesions of hypercolesterolemic *ldl*^−/−^ mice receiving anti-PD-1 antibodies (9), thus suggesting that andi-PD-1 drugs may result in an impairment of T-cell regulation leading to atherosclerotic plaque instability and rupture (21).

## Retrospective Studies

Only few retrospective studies have investigated the association between ICIs and AVEs (Table 3). In particular, an Israeli mono-institutional retrospective study on 1,215 cancer patients treated with ICIs from 2015 to 2018 reported 37 acute vascular events (3%), including cerebrovascular accident, transient ischemic attack, MI, ACS, embolic event, pulmonary emboli (21). In this study, the incidence of vascular events was significantly higher within the first 6 months (31 events, 1,215 patients at risk) than 7–12 months after ICIs initiation (6 events, 822 patients at risk), with an odd-ratio of 3.49 (95% CI 1.45–8.41, *p* = 0.002). Among the 31 patients with an early acute vascular event, 90% had ≥ 2 cardiovascular risk factors (smoking history, diabetes mellitus, hypertension, hyperlipidemia, male sex, past history of acute vascular events, and renal failure) and 55% had ≥3 risk factors. A multivariable analysis identified non-small cell lung cancer (NSCLC), history of acute vascular events and dyslipidemia as significant risk factors for AVEs during treatment with ICIs (23). Not unexpectedly, patients who developed an early vascular event had worse median overall survival (OS) than those who did not (3 vs. 14 months; HR 3.01, 95% CI 2.07–4.39, *p* < 0.0001), and in 25% of cases death occurred within 1 month from the vascular event.

**Table 3:** Retrospective studies.

**References**	**Study design**	***n***	**Main findings**
Gelsomino et al. (22)	Retrospective, mono-institutional	38	• 11 (29%) patients with atherosclerotic disease and complicated plaques at baseline • Of them, 3 patients (27.3%) had improvement, 7 patients (63.6%) had no changes, 1 patient (9.1%) had modest worsening of plaques after ICIs
Bar et al. (21)	Retrospective, mono-institutional, single cohort	1,215	• Incidence of AVEs within 6 months of ICIs: 2.6% (95% CI 1.8–3.6) • AVEs more frequent within 6 months than from 7 to 12 months of ICIs: OR 3.49 (95% CI 1.45–8.41, *p* = 0.002) • 90% of patients with AVEs had ≥2 CV risk factors • No difference in terms of response to ICIs or associated irAEs between pts who had or had not AVEs • Worse OS in pts with AVEs (3 vs. 14 months, HR 3.01, 95% CI 2.07–4.39, *p* < 0.0001)
Drobni et al. (23)	Retrospective, mono-institutional, matched 2-cohort study, with a case-crossover analysis and imaging sub-study	2,842 (ICIs)/2,842 (no ICIs)	• Matched cohort: higher risk of AVEs in ICIs vs. no-ICIs cohort (HR 3.3, 95% CI 2.0–5.5 *p* < 0.001) • Case-crossover: higher incidence of AVEs at 2 year after ICIs vs. 2 year before ICIs (adjusted HR 4.8, 95% CI 3.6–6.5, *p* < 0.001) • Imaging: Increased rate of progression of aortic plaque volume, from 2.1%/y before ICIs to 6.7%/y after ICIs

A matched cohort study of the Massachusets General Hospital included 2,462 cancer patients treated with ICIs from 2008 to 2012, and 2462 controls matched by age, history of cardiovascular events and cancer type, with the aim to evaluate whether exposure to ICIs was associated with AVEs defined as myocardial infarction, coronary revascularization and ischemic stroke (23). Results from this study showed that there was a 3-fold higher risk for AVEs after starting an ICI (HR 3.3, 95% CI 2.0–5.5; *p* < 0.001), in a multivariable model that included known cardiovascular risk factors (male sex, age, body mass index, hypertension, diabetes mellitus, chronic kidney disease, smoking, prior history of a CV event, statin use, aspirin use, hemoglobin, and low-density lipoprotein). Particularly, the use of ICIs was associated with a higher risk for MI (univariable HR 7.2, 95% CI 4.5–11.5; *p* < 0.001), coronary revascularization [univariable HR, 3.0 (95% CI, 1.9–4.8); *P* < 0.001], and ischemic stroke [univariable HR, 4.6 (95% CI, 2.9–7.2); *P* < 0.001] (23). In the same study, a case-crossover analysis of the cohort of patients treated with ICIs showed a significantly increased incidence of AVEs in the 2-year period after ICIs initiation compared to the 2-year period before (119 patients with AVEs, 4.2% vs. 66 patients with AVEs, 2.32%; HR 4.78, 95% CI 3.50–6.53, *p* < 0.001). Interestingly, in an imaging sub-study on 40 melanoma patients treated with ICIs, there was a >3-fold increase in the rate of atherosclerotic plaque progression after ICIs initiation (from 2.1% per year pre- to 6.7% per year post-ICI). As compared with non-statin users, patients receiving statins had lower yearly rates of plaque progression of total aortic plaque volume (5.2 vs. 8.3%, *p* = 0.04) and non-calcific plaque (3.1 vs. 7.0%, *p* = 0.04) (23).

In contrast with these results, a smaller retrospective study reported an improvement of atherosclerosis with nivolumab (22). Among 38 cancer patients included in the study, 11 had atherosclerotic disease with complicated aortic plaques at baseline. Of them, 3 (27.3%) showed a significant shrinkage of atherosclerotic plaques during nivolumab treatment, 7 (63.6%) had no significant changes and 1 (9.1%) had a modest worsening of the atherosclerotic lesions. Interestingly, one of the 3 patients achieving an atherosclerosis improvement, after intervening chemotherapy received subsequently the anti-PD-L1 antibody atezolizumab and again had a new reduction in aortic plaques until nearly complete resolution (22, 24). All the 3 patients with plaques reduction developed irAEs while on nivolumab, thus suggesting that the atherosclerosis improvement could have been related to a strong nivolumab-induced activation of their immune system. The biological mechanisms leading to the atherosclerosis improvement seen in this study are still unknown, but it has been hypothesized that PD-1/PD-L1 blockade may contribute to restore a protective role of T-cells on atheromatous plaques, impaired by plaque-associated macrophages and dendritic cells with hyperexpression of PD-L1. At this regard, a parallel histological study on archival surgical specimens of arteries with atherosclerotic lesions from non-cancer patients revealed that DCs with PD-L1 hyperexpression were observed in complicated plaques only, but not in early plaques (22). Therefore, it cannot be excluded that immuno-modulating agents such as ICIs could both promote or inhibit atherosclerosis. The reason why the pro-atherogenic or anti-atherogenic effect can prevail in the individual patient is unknown, but it possibly can involve several aspects of the plaque microenvironment including the severity of inflammation and the relative concentration of different cytokines. The plaque microenvironment could possibly vary not only between early and advanced plaques as demonstrated by histological studies (22), but also among different individuals and under different circumstances. A biological rationale for the atheroprotective effect of ICIs could be found in findings from a preclinical study reporting that PD-L1/PD-L2 deficiency in murine models may result not only in increased pro-atherogenic Th1 cells but also in increased anti-atherogenic Treg cells (10).

## Prospective Studies and Meta-Analyses

Data from prospective studies and meta-analysis suggested that AVEs are a rare event during treatment with ICIs. In fact, ICI-related AVEs have been only sporadically reported in prospective clinical trials. Particularly, few cases of MI were described in patients treated with atezolizumab for urothelial cancer (25) and with pembrolizumab for NSCLC (26).

In a meta-analysis evaluating the incidence of cardiovascular irAEs in cancer patients treated with ICIs, the incidence of MI was as low as 0.4% (95% CI 0.0–0.07%), although this result could be an under-estimation given that the 26 studies included were not specifically designed to evaluate the incidence of cardiovascular toxicity and only 6 out of 26 reported the incidence of MI as an irAE (27). Similarly, another meta-analysis reported a low rate also for arterial thromboembolic events (1.1%, 95% CI 0.5–2.1%) among over 20,000 cancer patients treated with ICIs in 68 studies (Table 4) (28).

**Table 4:** Meta-analyses.

**References**	***N* patients (*n* studies); cancer**	**Main findings**
Nso et al. (27)	4,622 (26); various cancers	• Incidence of MI: 0.4% (95% CI 0.1–0.8%)
Solinas et al. (28)	20,273 (68); various cancers	• Incidence of arterial thromboembolic events: 1.1% (95% CI 0.5–2.1%) • Incidence of stroke: 1.1% (95% CI 0.65–1.45%) • Incidence of MI: 0.7% (95% CI 0.15–1.15%),
Hu et al. (29)	4,828 (22), NSCLC	• Incidence of MI: 1.0% (95% CI, 0–3.8%) • Incidence of stroke: 2.0% (95% CI, 0–13.0%)

The primary site of cancer may represent a risk factor itself for the development of AVEs. As reported before, patients with lung cancer treated with ICIs seem to have higher incidence of AVEs. In fact, a meta-analysis of 22 trials on NSCLC patients treated with ICIs reported an 1.0% incidence rate of MI (95% CI, 0–3.8%) and 2.0% of stroke (95% CI, 0–13.0%) (29). Consistently with this report, a *post-hoc* analysis of a prospective observational study reported high incidence of arterial thromboembolic events among 217 NSCLC patients treated with ICIs (16 events, 6.5%) (30). Interestingly, in this study patients receiving antiplatelet treatment experienced longer progression-free survival (6.4 vs. 3.4 months; HR 0.67, 95% CI 0.48–0.92; *p* = 0.015) and a trend toward better OS (11.2 vs. 9.6 months; HR 0.78, 95% CI 0.55–1.09; *p* = 0.14) (30).

## Discussion

In prospective clinical trials and meta-analysis, the incidence of AVEs during treatment with ICIs was relatively low (31). However, because AVEs have not been typically considered as irAEs, until recently they could have been under-reported in clinical trials and, consequently, also in meta-analysis. Therefore, their actual incidence could be under-estimated (32). The same has already happened for other ICI-related cardiovascular toxicities, such as myocarditis. Immune-related myocarditis, in fact, has been under-reported until 2016, when two cases of fulminant myocarditis were described (33). Since then, the reporting of such events has been increasing, possibly due to increased awareness among clinicians and more detailed cardiac assessments detecting evidence of milder cardiovascular toxicity (31).

Patients enrolled in clinical trials are usually a highly selected population, and elderly patients who may have subclinical atherosclerosis, as well as those with high cardiovascular risk or history of cardiovascular disease, have been often excluded or under-represented in clinical trials investigating ICIs (21, 23). This argument could contribute in part to explain why the incidence of AVEs is low in prospective clinical trials, but becomes meaningfully higher in real-word retrospective studies (3–4%) enrolling patients with higher cardiovascular risk (21). It is known that cardiovascular risk factors, particularly dyslipidemia and history of acute vascular events, may increase the risk for AVEs among cancer patients treated with ICIs, as clearly showed by real-word evidence (23).

In a recently published, well-designed matched cohort retrospective study, treatment with ICIs significantly increased the risk for AVEs and the atherosclerotic plaques volume. This finding is consistent with preclinical data showing that CTLA-4 and PD-1 blockade accelerates the progression of atherosclerotic plaques (22, 24). However, there is also conflicting evidence deriving from a smaller retrospective study that suggested instead an atheroprotective role for anti-PD-1/PD-L1 agents (34, 35). These contrasting observations underline that the interactions among cancer, atherosclerosis, and immune system are still far from being comprehensively understood, therefore further basic and clinical research in this field is urgently needed. The possible role of the microenvironment in modulating the adaptive immune response within the atherosclerotic plaques may offer interesting insights for research, since strategies aiming to manipulate the plaque microenvironment may potentially improve the cardiovascular safety profile of ICIs.

The research on the correlation between ICIs and AVEs is now particularly important, since several combinations of ICIs with other drugs such as anti-angiogenesis agents, that potentially increase the risk for arterial thrombosis and acute vascular events, have been recently introduced in clinical practice (36). Although clinical trials investigating these combinations did not report a significant excess of AVEs, it should be kept in mind that atherosclerosis-related complications may develop gradually over years or even decades, and the post-marketing surveillance is still limited to have sufficient data on long-term adverse events. Moreover, beyond anti-CTLA-4 and anti-PD-1/PD-L1 antibodies, different ICIs are currently under clinical investigation, including antibodies directed against checkpoints that may have a role in regulating the atherosclerosis process and maintaining cardiovascular homeostasis, such as the T-cell immunoglobulin and mucin-domain (TIM) proteins, T cell immunoglobulin and ITIM domain (TIGIT), and OX40 (23, 30).

Some prospective studies designed with the aim to collect data on AVEs and other cardiovascular toxicities among cancer patients receiving ICIs are currently ongoing (NCT04586894, NCT03709771, NCT04115410), and their results will probably provide better knowledge on the correlation between ICIs and AVEs. However, research efforts should be also directed to translational studies aiming to identify novel circulating biomarkers or possibly immunogenomic factors that may predict for cardiovascular toxicity of ICIs (37).

Taken into account the available evidence, it would be advisable that cancer patients who are candidates to receive ICIs are carefully assessed for known cardiovascular risk factors based on easy-to-use scoring systems such as the Systemic Coronary Risk Estimation (SCORE) (37). Baseline assessment and periodical monitoring of body weight, blood pressure, cholesterol, and glycemia should be performed in all cancer patients receiving ICIs. An optimization of cardiovascular risk factors and medical therapy for primary or secondary prevention before, during and after ICIs should be considered. Patients should be always supported for smoking cessation and adoption of healthy lifestyle and healthy diet, although it can be often difficult for cancer patients, especially those with metastatic disease, to do regular physical activity or follow a strict diet. In addition to behavior changes, medical therapy such as lipid-lowering drugs, blood pressure-lowering drugs, oral blood glucose-lowering drugs or insulin, and anti-platelet agents should be appropriately used to manage cardiovascular risk factors including dyslipidemia, diabetes mellitus and hypertension, according to well-established guidelines (37).

This approach will require ever closer cooperation between oncologists and cardiologist in the near future.

## Author Contributions

AI wrote the draft. All authors critically revised and approved the manuscript.

## Conflict of Interest

The authors declare that the research was conducted in the absence of any commercial or financial relationships that could be construed as a potential conflict of interest.
